# Unlocking the Potential: Increasing Muscle Strength in Lower Limbs of Youth Soccer Players over Five Weeks through Mat Pilates Training—A Pilot Study

**DOI:** 10.3390/s24020473

**Published:** 2024-01-12

**Authors:** Franciele Parolini, Gladson Bertolini, Rubim Santos, Manoela Abreu, Ana Laura Nogueira, Dernival Bertoncello

**Affiliations:** 1 Human Movement Analysis Laboratory (LAHM), Federal University of Triângulo Mineiro (UFTM), Av. Getúlio Guaritá, 159, Nossa Sra. da Abadia, Uberaba 38025-440, MG, Brazil; fcsp@ess.ipp.pt (F.P.); manuh-abreu94@hotmail.com (M.A.); analaura_fisio@hotmail.com (A.L.N.); dernival.bertoncello@uftm.edu.br (D.B.); 2 Center for Rehabilitation Research (CIR), School of Health, Polytechnic Institute of Porto, Rua Dr. António Bernardino de Almeida, 400, 4200-072 Porto, Portugal; 3 Center of Research, Education, Innovation and Intervention in Sport, Faculty of Sport, University of Porto, 4200-450 Porto, Portugal; 4 Porto Biomechanics Laboratory (LABIOMEP), University of Porto, 4200-450 Porto, Portugal; 5 Department of Physiotherapy, State University of Western Paraná (UNIOESTE), Cascavel 85819-110, PR, Brazil; gladsonricardo@gmail.com

**Keywords:** mat Pilates, sports, instrumentation, biomechanics, youth soccer players, training

## Abstract

The interest in soccer generally starts during childhood, with children and young people often looking for opportunities in sports. New exercise techniques can be effective in improving training. The aim of this study was to compare the effects on the strength and physical posture of a group practicing Pilates with another not practicing Pilates, both undergoing continuous football training. In this controlled randomized clinical trial, the participants were 15 soccer club members, who had a training frequency of least three times weekly. The sample was divided into a control group (*n* = 7) of players who did not undergo any therapeutic intervention (only the usual training) and a Pilates group (*n* = 8) of players who participated in the mat Pilates program. The intervention consisted of fifteen sessions. Postural evaluations were performed using biophotogrammetry and force analysis. Significant improvements were obtained in terms of increased muscle strength (*p* = 0.001) for the Pilates group, but there were no significant postural alterations when comparing the two groups. Five weeks of mat Pilates was sufficient to increase lower limb muscle strength in young football players. This pilot study indicates that Mat Pilates as a method that could be planned to be included in training.

## 1. Introduction

The enthusiasm for soccer typically begins in early childhood worldwide, with children and adolescents frequently exploring prospects as sports professionals [1]. Engagement in the sport has been facilitated by improvements in access to scholastic sports activities, recreational games, and competitive training teams [2]. Within the realm of football activity, participants are required to exert significant physical effort, often approaching the limits of exhaustion. They must bear the weight of their bodies and execute movements in diverse directions and manners, marked by rapid and improvised positional changes during both training sessions and matches [3,4]. The nature of football activity requires specific characteristics, as the expansive playing field demands heightened physical prowess, particularly in running. Meanwhile, adept footwork necessitates well-honed technical and tactical capabilities. Players engage in four primary running modes: slow, submaximal speed, maximum speed, and backward running.

Consequently, individual physical preparation should focus on specific exercises that elicit anatomical and physiological adaptations tailored to the requirements of this sport [5]. The initiation of sports practice occurs at an increasingly young age, underscoring the necessity for the training of young soccer athletes to consider their biological maturity in relation to physical performance to avert adverse effects [6,7]. Mitigating potential postural effects involves implementing practical training programs designed to systematically cultivate behavioral skills and augment knowledge, thereby benefiting players’ habits and health [7,8]. However, the literature lacks clinical trials that provide evidence on the effects of low-cost and easily accessible training programs that are effective for the under-13 and under-15 soccer categories. Pilates emerges as a viable option that could be recommended as a complementary method to strengthen the muscles that contribute to the to body posture and, consequently, enhancing athletes’ performance, particularly during the growth phase [9,10,11,12]. It would make an important contribution to trunk stabilization and allow children to perform better in sports by working deeper muscles. As the muscles and bones are in the formation phase, the correct stimulus could lead to the best static body posture and for sporting gestures [9,10,11,12].

The controlled and precise performance of mat Pilates exercises induces muscle strengthening, enhancing the strength, endurance, and flexibility of the involved muscles. These improvements can be attributed to structural and functional changes in the muscle sarcomeres, as well as modifications in the biomechanical properties of tendons and ligaments [13,14,15]. The emphasis on motor control during Pilates practice plays a crucial role in this process, inciting neural plasticity and facilitating refined coordination between cortical motor centers and peripheral motor units [15,16,17,18]. This phenomenon leads to notable enhancements in the efficiency of motor patterns and the optimization of neuromuscular responses, resulting in joint stability and precise alignment. The synchronization of breathing with movements is a distinctive feature of Pilates [12,19,20,21]. This component contributes not only to biomechanical efficacy but also to trunk stabilization, enhancing movement efficiency and promoting body awareness [10,22,23,24,25]. Such an approach contributes to the proportional development of muscle strength and improved body awareness, positively affecting proprioception and postural control.

The present study focuses on integrating the Pilates method into the regular physical training regimen of youth soccer players, a context where personalized training is not commonly implemented. The objective was to assess the impact on strength and physical posture in a group practicing Pilates compared to another group not incorporating Pilates, both undergoing continuous football training [12,26]. The underlying hypothesis posited that Pilates training would lead to improved body posture and enhanced lower limb strength in athletes. Specifically, Pilates training was expected to strengthen the deeper core muscles, consequently improving body posture and augmenting lower limb strength in athletes, potentially contributing to enhanced sports performance.

## 2. Materials and Methods

The current investigation adhered to the standards for conducting research involving human subjects, following the guidelines outlined in Resolution 196/466 of the National Health Council concerning research with human participants. Approval for the study was obtained from the Research Ethics Committee of the University (protocol 2,827,678), granted in 2018, and the study is duly registered with the Brazilian Registry of Clinical Trials (ReBEC) under the identifier RBR-6Z2DHD PILATES IN SOCCER. Before providing formal consent to participate, all participants (and their legal guardians) received comprehensive information on the study’s nature.

### 2.1. Sample

Invited participants in this study consisted of 30 soccer players aged between 13 and 14 years, affiliated with the Uberaba Sports Club. The primary objective of this study aligns with that of pilot studies. In a convenience sample, all club players meeting the inclusion criteria were invited to participate. Nineteen players voluntarily consented, each possessing a minimum of five years of competitive experience and engaging in training sessions at least five times per week, each lasting four hours. The assessment included postural aspects of the lower limbs, as well as the strengths of knee extensors and flexors, and abdominal muscles. Participants were thoroughly briefed on the study’s objectives and methodology, and their legal guardians provided free and informed consent.

The exclusion criteria for this study were as follows: athletes with a training frequency of less than three times weekly, a weekly training load of fewer than nine hours, a history of muscle injury during the intervention and/or evaluation period, orthopedic surgery in the knee, ankle, or hip during the assessment and/or intervention period, and failure to attend evaluations and/or interventions for two consecutive days.

Following these criteria, 15 athletes meeting the specified conditions were identified and randomly assigned to either a Pilates group (PG) or a control group (CG). The allocation process utilized a software tool that generated random numbers, with participants receiving odd numbers being assigned to the Pilates group (*n* = 8) and those receiving even numbers being assigned to the control group (*n* = 7). Both the PG and CG players demonstrated homogeneity in terms of age, weight, and body mass index (*p* > 0.05).

### 2.2. Experimental Design

In this study, the two groups participated in activities on separate days, ensuring a minimum interval of 48 h between sessions. The initial laboratory session included the signing of free and informed consent forms, followed by anthropometric assessment, postural and strength analyses, and familiarization with the equipment. Administered by professionally trained physiotherapists, the Pilates exercises were conducted, and evaluations were carried out both before and after the intervention period. Comprehensive training was provided to the professionals, addressing in detail the evaluation criteria and the specific methods to be employed. Notably, the same professional conducted assessments for all participants at both time points. This training involved hands-on sessions, a thorough review of protocols, and clarification of any doubts that arose. We aimed to mitigate potential sources of error by ensuring that assessments are reliable, consistent, and capable of providing valid data throughout all phases of the study. The postural analysis of the lower limbs occurred in the Human Movement Analysis Laboratory (LAMH) at the Federal University of Triângulo Mineiro (UFTM). Volunteers were assessed in three groups, each comprising 5 athletes, on alternate days, starting four weeks before the initiation of the Pilates intervention.

### 2.3. Photo Registration

Photographs were captured following the recommendations of the SAPO© software, version 0.69. A plumb line was affixed to the ceiling, and two polystyrene balls, spaced 1 m apart, were attached to the plumb line for subsequent image calibration. The object was positioned to ensure that the object and plumb line were within the same plane perpendicular to the axis of the digital camera (Canon EOS 4000D + EF-S 18–55 mm f/3.5–5.6 DC II, with a resolution of 15.0 megapixels). The camera was placed 3 m away and mounted on a tripod positioned approximately halfway to the subject’s height [27]. The volunteers were photographed in anterior, posterior, right lateral, and left lateral views, all in the anatomical position.

**Figure 1:**
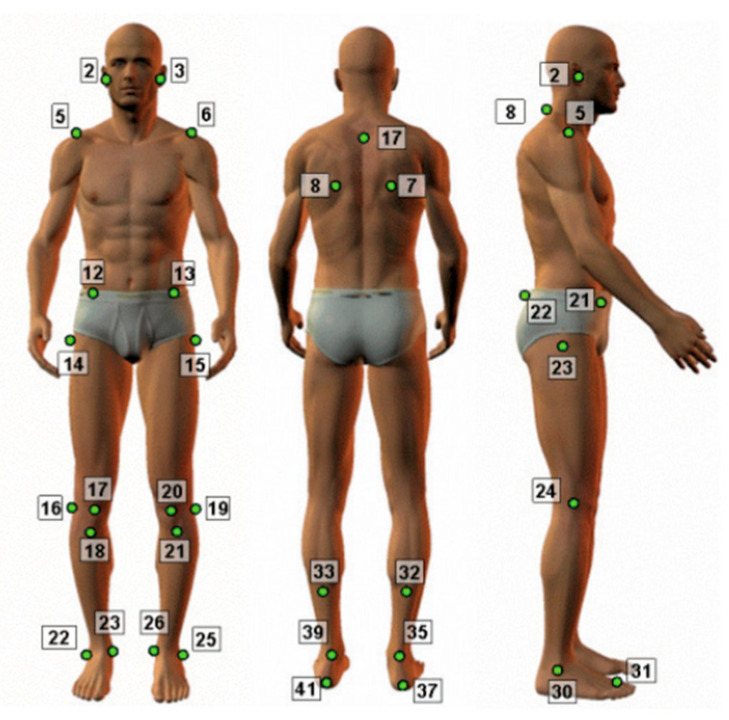
Bone references from the SAPO© software protocol [28].

The bone references, utilized as guides for angle calculations, were marked with polystyrene spheres following the SAPO© protocol, at anatomical points in the anterior view (2, 3 right and left tragus; 5, 6 right and left acromion; 12, 13 right and left anterosuperior iliac spine; 14, 15 right and left greater trochanter; 16, 19 lateral projection of the right and left knee joint line; 17, 20 center of right and left patella; 18, 21 right and left tibial tuberosity; 22, 25 side malleoli; 23, 26 medial malleoli); posterior view (7, 8 lower angle of the right and left scapula; 17 third thoracic vertebra; 32, 33 medial point of the leg; 35, 39 intermalleolar line; 37, 41 calcaneal tendon bilaterally); and lateral view (2 tragus; 8 seventh cervical vertebra; 5 acromion; 21 anterosuperior iliac spine; 22 posterior superior iliac spine; 23 greater trochanter; 24 projection of the knee joint line; 30 lateral malleolus; 31 region between the second and third metatarsals) [27,28], described in Figure 1.

### 2.4. Photogrammetry

The photographs were transferred to a computer, and subsequently, copies were provided to two examiners proficient in the SAPO© program for photometric analysis of the subjects’ body postures [28,29]. During the analysis of the photos, the following steps were undertaken: image correction, marking of protocol points, generation of an analysis report, and export to Excel. The angles of the protocol are described in Figure 1. The marking of the anatomical points and the imaging exams were consistently conducted by two expert reviewers. The quantification of the angle between the anatomical points, following the protocol, was automatically generated and adheres to the conventions of the program.

### 2.5. Isometric Muscle Strength Assessment

For the isometric assessment of knee extensors and flexors in the dominant limb, a manual dynamometer from Lafayette^®^—model 01165—was employed [30]. Volunteers were seated at a table for the knee extensor test and in a prone position for the knee flexor test, and were stabilized on the table with nylon straps, a resin-coated, sturdy material used to secure the hip. The knee flexor test was conducted with the knee in an extended position, and the dynamometer was positioned anteriorly to the ankle, 5 cm above the lateral malleolus. For the knee extensors, the test was conducted with the knee at a 90° angle, and the dynamometer was positioned posteriorly at the calcaneus region [30,31,32].

For the assessment of anterior trunk flexors, participants were positioned lying on a dorsal decubitus bed, with the dynamometer placed between the resistance band and the upper sternal region [33]. Resistance during the strength test was applied using the same nylon band as in previous tests, fixed to the bed and adjusted to each participant’s trunk. A second band was placed on the distal quadriceps region to stabilize them on the bed. This band was used to eliminate potential oscillations when manual resistance was applied by the examiner on the dynamometer. Thus, the examiner solely stabilized the dynamometer, preventing possible displacements under the band. Prior to the tests, a maximum isometric contraction was requested for each muscle group to familiarize participants with the procedures and equipment. During this phase, participants received instructions on the positioning and execution of each test, ensuring their comprehension of the testing procedure [34].

Following this process, three maximum isometric contractions were requested, and the mean force between the three measurements was considered for analysis. The dynamometer was zeroed before each isometric contraction, and the maximum force value was recorded for each contraction. The duration of each contraction was standardized at 5 s, followed by 30 s of rest. Data were recorded in newtons (N). If a variation greater than 10% occurred between the peak maximum force obtained in the three attempts, a fourth attempt was conducted [35]. In such cases, the peak force of the three attempts with the least variation among them was used for analysis.

### 2.6. Mat Pilates

**Figure 2:**
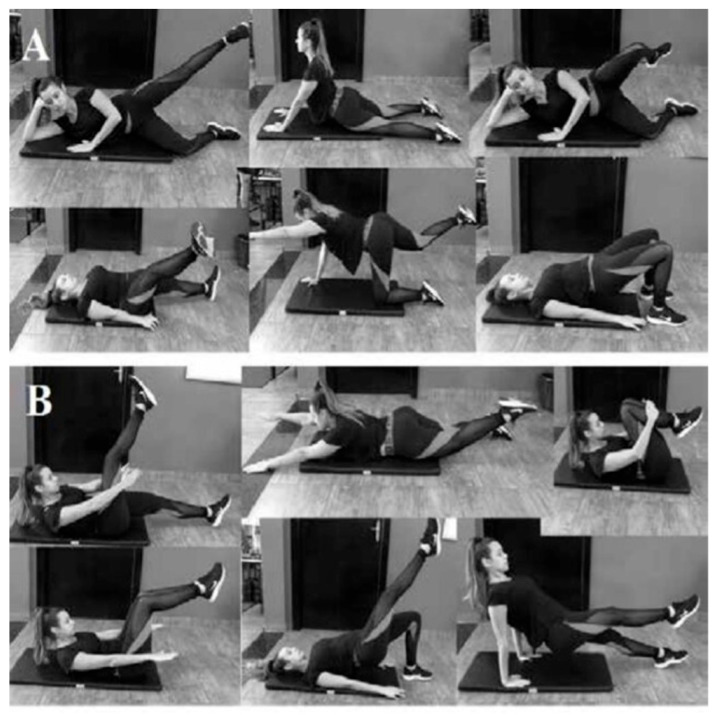
The sequence of exercises of Pilates. (**A**) Basic exercises above, in order: Side kick; Shoulder bridge; Clamshell; One Leg Circles; Quadruped; The Swan Dive. (**B**) Intermediate exercises above, in order: Scissor; Swimming; one-leg Shoulder bridge; Hundred; The Double Leg Stretch; Leg Pull.

Ten different Pilates exercises were performed, each consisting of sets of ten repetitions. These exercises were selected based on their benefits in terms of flexibility, muscular strength, body awareness, and balance. The volunteers were provided instructions on adhering to the principles of the Pilates method (Center, Control, Concentration, Fluidity, Precision, and Breathing) during the execution of each exercise [25]. The interventions were supervised by a certified Pilates practitioner with experience in the method. The exercises, listed in Table 1, were as follows:

The following repetitions of each exercise were performed: Basic (A): side kick (10 repetitions each leg); shoulder bridge (10 repetitions); one leg circles (10 repetitions); clamshell exercise (10 repetitions); Intermediate (B): scissor (10 repetitions each leg); swimming (10 repetitions); shoulder bridge unipodal (10 repetitions each leg); hundred (10 series with 10 pumping movements); double leg stretch (10 repetitions); leg pull (10 repetitions), demonstrated in Figure 2 [36,37].

The protocol with the PM was carried out in five weeks, three times a week, lasting 30 min, totaling 15 sessions [26]. The first week was for adaptation, starting one week after the assessment; the second and third weeks consisted of basic exercises and the fourth and fifth weeks, intermediate exercises. The protocol consisted of 12 exercises with ten repetitions each, aiming at flexibility, improving muscle strength, body awareness, and balance. The interventions were directed by professionals with experience in the method.

### 2.7. Statistical Analysis

The results were presented as means with standard deviations and delta values. Normality was assessed using the Shapiro–Wilk test. To examine potential differences between the results of the evaluations conducted before and after the intervention, the student’s t-test was employed for normally distributed samples (data for knee flexors and extensors, anterior superior iliac crest (ASIC), and right and left ankles). For non-normally distributed samples (data for anterior trunk flexors and left and right knees), the Wilcoxon–Mann–Whitney test for independent samples was utilized. The significance level adopted was set at *p* < 0.05. The Cohen’s D test was employed to assess the effect size. We considered Cohen’s D from 0.2 to 0.5 as small, from 0.5 to 0.8 as moderate, and values above 0.8 as large. The statistical procedures were performed using the Statistical Package for the Social Sciences (SPSS, version 26.0 for Windows).

## 3. Results

Among the 15 athletes, eight initiated mat Pilates classes, and all eight successfully completed the intervention, totaling 15 sessions. The subjects’ mean age, height, and body weight are detailed in Table 2. Importantly, no statistically significant differences were observed between the control and Pilates groups regarding these variables. Consequently, it can be asserted that there is homogeneity between the experimental and control groups, rendering them comparable.

The results of the strength test applied to the following muscle groups: knee flexors and knee extensors, before and after mat Pilates (Table 3), demonstrated significant increases (*p* = 0.0001). The intra-group comparison also revealed improvements in the control group, but with higher coefficients compared to the Pilates group. Cohen’s D test values ranged from high to very high, indicating that the greater the difference between the means of the treatments detected, the greater the effect of the sample size, with a smaller sample size being associated with a greater effect [38].

Table 4 presents the postural evaluation of the athletes. The measurements of the right and left knee Q angles, the right and left ankle angles, and the anterior superior iliac spine showed no significant differences (*p* = 0.862). The intra-group analyses indicated that both the Pilates and control groups did not exhibit any significant changes in terms of the postural parameters, thus maintaining the characteristics observed in the initial evaluations. The Cohen’s D test values were not significant, likely due to the small differences between the means for the treatments [38].

## 4. Discussion

The primary objective of the current study was to examine the impact of a concise Pilates protocol on lower limb strength and body posture in young soccer athletes. Our initial hypothesis was substantiated, as evidenced by an increase in isometric strength observed in certain muscles compared to athletes in the control group. Beyond the evaluation of muscle strength, an equally significant consideration is the assessment of range of motion, particularly pertinent for soccer athletes. The emphasis lies in establishing an association between diverse variables to ensure optimal mobility and heightened joint stability, with the overarching goal of injury prevention and facilitating enhanced athletic performances [34,39].

The assessment of postural and strength alterations in the lower limbs and trunk, conducted before and after 15 sessions of the mat Pilates method, aimed to gather insights that could contribute to the prevention of future injuries. Notably, during the intervention period applied in this study, no discernible postural changes associated with the application of the mat Pilates method were observed in the subjects. This finding contrasts with previous research; for instance, Segal et al. [40] and Donahoe-Fillmore et al. [41] reported that a Pilates protocol administered once a week for one hour over two months resulted in enhanced joint flexibility, albeit with no discernible changes in body composition (weight and posture). There would possibly be a need for more training time for these athletes to verify notable postural changes [42].

In a comprehensive systematic review by Cruz-Ferreira et al. [43] addressing exercise programs targeting injury prevention in soccer players, 23 studies were considered. In nearly all instances, a singular exercise program was implemented as an intervention alongside regular training, except for one study. Intriguingly, none of the reviewed studies explored the potential benefits of the Pilates method in soccer, underscoring the prevailing trend of employing Pilates primarily for rehabilitation rather than as a proactive strategy for injury prevention. The current study marks an innovative departure by introducing Pilates specifically for young football athletes in the context of injury prevention.

It is plausible that the duration of Pilates training in our study might have been insufficient to yield significant improvements in posture. Achieving notable changes may necessitate further muscle strengthening, influencing the regions where muscles originate and/or insert, consequently modifying bone structure and rectifying posture. Johnson et al. [44] proposed that Pilates exercises challenge sensory systems responsible for balance and dynamic postural control. The effects, particularly the strengthening of core muscles, may become apparent after a mat Pilates program comprising more than 20 sessions. In line with this, Rossi et al. [45] conducted a randomized controlled trial involving 33 volunteers, revealing postural adaptations following twice-weekly Pilates exercises over ten weeks.

In the current study, the 15 sessions of mat Pilates did not induce sufficient adaptations to generate discernible differences in the static postural alignment of soccer players. Consistent with prior research, it appears that the effects of exercises focusing on postural reeducation may be time-dependent, necessitating long-term structural adaptation and the acquisition of body postural awareness [20]. Highlighting the potential of proprioceptive muscle training, demonstrated its capacity to improve dynamic postural responses. Therefore, future studies, in addition to assessing static postural alignment, should consider functional variables related to dynamic postural balance [46]. According to Cruz-Dias [21,45], the comparative effects of Pilates performed using apparatus versus mats warrant exploration, ideally with large sample sizes, to provide a comprehensive understanding of the impact of different Pilates modalities on postural outcomes.

Contrastingly, mat Pilates resulted in significantly higher values for muscle strength. In essence, the mat Pilates method does not primarily aim to induce hypertrophy, a characteristic observed in other methods utilizing load to recruit and increase muscle fiber volume [47]. The method is characterized as a complement to hypertrophy’s role in augmenting muscle mass, with mat Pilates exercises emphasizing the quality of movement over the quantity of repetitions performed [22,48]. Therefore, as anticipated, the intervention did not exert a substantial influence in instigating new muscle trophism. Nonetheless, it holds potential significance in terms of motor control [24].

Muscle, as an organ, has the capacity to produce hormonal growth factors such as IGF and MGF (insulin-like and mechanic growth factors) when subjected to submaximal contraction [49]. This process facilitates the activation of muscle satellite cells, promoting protein synthesis through molecular pathways, ultimately resulting in an increase in muscle volume, commonly referred to as hypertrophy [37]. Notably, exercises within the mat Pilates method involve motor activity, indicating the presence of mechanical stimulation at the myotendinous junction [22,49].

In a study conducted by Oliveira et al. [16], the effects of Pilates practice on neuromuscular adaptations and body composition were analyzed in ten young adults of both sexes. While measurements of body composition did not exhibit significant differences, noteworthy effects were observed in terms of performance and muscle strength. As highlighted, Pilates training yields important positive outcomes in balance, coordination, flexibility, and breathing, achieving enhanced strength with a relatively smaller muscle volume [23,50].

The present study’s findings demonstrated that mat Pilates could contribute to increased strength in the muscles of the lower limbs and trunk, with this effect becoming apparent within a five-week training period. The observed strength increase may be attributed to the recruitment of a greater number of motor units, without necessarily implying hypertrophy. This suggests that muscles began to function more efficiently, involving a heightened number of active motor units [36,37]. However, there are limitations. As our study is a pilot, the main focus was on verifying whether there would be a contribution to increased strength after Pilates exercises. The absence of significant postural changes after the five-week intervention period raises considerations about the timing of these adaptations, indicating that the impact of Pilates on posture may require a more extended training duration. This conclusion aligns with the existing literature, underscoring the importance of time in achieving substantial postural benefits. It can be asserted that the results of this study offer significant contributions to both health professionals/coaches and other sports practices.

However, the study is not without limitations. The relatively small number of volunteers, despite the inclusion of all team members from the chosen club, is acknowledged. Additionally, the study’s restriction to a limited number of Pilates training sessions suggests a potential avenue for future research, encouraging investigations with an extended duration of training and long-term monitoring for more comprehensive insights. Increasing the sample size and extending the duration or frequency of sessions could provide a more comprehensive and robust view of the benefits of the method, especially in the context of injury prevention and improvement in joint stability. Future studies will have the ability to offer a deeper and more reliable understanding of the effects of mat Pilates and machine-based Pilates on postural changes, thus contributing to the evolution of knowledge in this area. Another perspective would be to conduct further studies to examine how Pilates could improve muscle activation to the point of modifying the muscle synergy patterns of athletes’ lower limbs and consequently improving their general performance.

## 5. Conclusions

In summary, the results of this study highlight the effectiveness of a 4-week mat Pilates program in improving muscle strength in the lower limbs and trunk of soccer players in the under-13 category, compared to the control group. However, it is crucial to note that this relatively short period was not sufficient to induce significant postural changes. The emphasis on enhancing muscle strength, especially in the lower limbs and trunk, suggests that mat Pilates could be a valuable addition to the training of young soccer players, providing a cost-effective and easily accessible option for all clubs, potentially contributing to enhancing sports performance and preventing injuries.

## Figures and Tables

**Table 1:** Exercises and objectives for mat Pilates.

Exercise | Level | Week	Objective
Side Kick | B | 1° and 2° week	To work the Powerhouse, improve the strength and flexibility of the hip, quadriceps, and abdominal muscles, and train motor control.
Shoulder Bridge | B | 1° and 2° week	To improve mobility of the thoracic and lumbar spine and strengthen glutes and hamstrings.
One Leg Circles | B | 1° and 2° week	To work on the mobility of the hip joint, strengthen the buttocks, and elongate the iliotibial band.
Clamshell Exercise | B | 1° and 2° week	Strengthen the gluteus medius and improve hip mobility.
Scissor | I | 3° and 4° week	To work pelvic and scapular stability and flexibility of the hip flexors, focusing on controlled movement by the Powerhouse.
Swimming | I | 3° and 4° week	To improve hip mobility, strengthen the spine, and enhance movement mechanics such as walking or running.
Shoulder Bridge Unipodal | I | 3° and 4° week	To mobilize the spine, train the interaction among hip, ribs, and the scapular girdle, and strengthen the glutes and hamstrings.
Hundred | I | 3° and 4° week	To strengthen the abdomen and the Powerhouse, improving balance and power output.
Double Leg Stretch | I | 3° and 4° week	To work the Powerhouse, pelvic alignment, and leg stretching.
Leg Pull | I | 3° and 4° week	To improve hip mobility, strengthen the spine, and maintain pelvis and spine stabilization.

Legend: basic exercise level (B), intermediate (I), duration of application of the weekly exercise (week).

**Table 2:** Sociodemographic characteristics of participants.

Variables	Total	Pilates Group	Control Group	*p*
Age (years)	13.2 ± 0.4	13.2 ± 0.4	13.2 ± 0.4	0.8
Mass (kg)	51.9 ± 5.9	54.3 ± 5.9	49.2 ± 5.2	0.8
Height (cm)	164.2 ± 5.3	164.5 ± 4.6	164.0 ± 6.4	0.1
BMI (kg^2^/m^2)^	19.1 ± 2.3	19.8 ± 2.2	18.3 ± 2.4	0.3

**Table 3:** Muscle strength in newtons (N) from an evaluation of the Pilates and control groups, before and after the intervention.

Variables	Group	Before	After	*p*	Delta	*p* (dif.)	Cohen’s D
Knee Extensors	Control Pilates *	73.6 ± 8.3 72.1 ± 8.2	74.9 ±11.8 89.7 ± 7.6	0.656 <0.001	1.2 ± 7.8 17.5 ± 6.6	0.001	2.28
Knee Flexors	Control Pilates *	58.1 ± 12.9 56.2 ± 10.0	54.0 ± 13.3 63.1 ± 7.2	0.041 0.016	−4.1 ± 4.6 6.9 ± 5.5	0.001	1.45
Trunk flexors	Control Pilates	54.4 ± 8.5 49.2 ± 14.1	47.1 ± 3.0 51.3 ±13.7	0.036 0.609	−7.3 ± 8.0 2.0 ± 10.0	0.011	1.06

Legend: mean ± standard deviation, *p*-value, delta value (mean ± standard deviation), difference *p*-value, * difference significative, and Cohen’s D test value.

**Table 4:** Anatomic angles (in degrees) for the Pilates and control groups, before and after the intervention.

Variables	Group	Before	After	*p*	Delta	*p* (dif.)	Cohen’s d
ASIC	Control Pilates	0.41 ± 2.00 0.04 ± 1.83	0.56 ± 1.79 0.70 ± 0.59	0.780 0.420	0.15 ± 1.4 0.65 ± 2.1	0.862	−0.06
Knee R	Control Pilates	11.5 ± 8.30 13.4 ± 4.60	11.1 ± 7.60 12.9 ± 2.90	0.696 0.696	0.38 ± 2.6 0.56 ± 3.6	0.914	−0.10
Knee L	Control Pilates	11.8 ± 8.60 13.5 ± 4.60	11.8 ± 8.00 12.9 ± 2.84	0.981 0.633	0.02 ± 2.8 0.6 ± 3.20	0.711	−0.04
Ankle R	Control * Pilates	82.7 ± 7.90 75.3 ± 8.10	85.0 ± 4.30 83.1 ± 4.40	0.357 0.028	2.2 ± 6.40 7.8 ± 6.90	0.134	−0.01
Ankle L	Control Pilates	84.6 ± 4.80 79.6 ± 7.20	84.8 ± 4.20 82.5 ± 4.20	0.894 0.176	0.2 ± 4.60 2.9 ± 4.70	0.289	−0.01

Legend: anterior superior iliac crest (ASIC), knee right (R), knee left (L), ankle right (R), ankle left (L), mean ± standard deviation, *p*-value, * difference significative, delta value (mean ± standard deviation), difference *p*-value, and Cohen’s D test value.

## Data Availability

The data presented in this study are available on request from the corresponding author. The data are not publicly available due to privacy.
